# Education and indoor smoking among parents who smoke: the mediating role of perceived social norms of smoking

**DOI:** 10.1186/s12889-018-5082-9

**Published:** 2018-02-02

**Authors:** Junko Saito, Akira Shibanuma, Junko Yasuoka, Naoki Kondo, Daisuke Takagi, Masamine Jimba

**Affiliations:** 1 Department of Health and Social Behavior, Department of Health Education and Health Sociology, School of Public Health, The University of Tokyo, 7-3-1 Hongo, Bunkyo-ku, Tokyo, 113-0033 Japan; 2 Department of Community and Global Health, Graduate School of Medicine, The University of Tokyo, 7-3-1 Hongo, Bunkyo-ku, Tokyo, 113-0033 Japan; 3 Research and Education Center for Prevention of Global Infectious Diseases of Animals, Tokyo University of Agriculture and Technology, 3-8-1 Harumi-cho, Fuchu-shi, Tokyo, 183-8358 Japan; 4 Department of Health and Social Behavior, School of Public Health, The University of Tokyo, 7-3-1 Hongo, Bunkyo-ku, Tokyo, 113-0033 Japan

**Keywords:** Tobacco smoke pollution, Educational status, Social norms, Fathers, Mothers

## Abstract

**Background:**

Parents with less formal education are more likely to smoke indoors, causing socioeconomic disparity in children’s exposure to second-hand smoke. However, little is known about the roles of social factors in the socioeconomic gradients of indoor smoking. We tested the potential mediating role of perceived smoking norms on the associations between education and indoor smoking among parents who smoke.

**Methods:**

In this cross-sectional study, 822 smoking fathers and 823 smoking mothers, who lived with young children and were members of a Japanese online survey panel, participated. Structural equation modelling tested the mediating effects of perceived descriptive and subjective norms on the association between education and indoor smoking.

**Results:**

Perceived pro-smoking norms, which were more prevalent among less-educated parents, mediated the association between education and indoor smoking. Household smoking status and worksite smoking ban also mediated this association via perceived norms, but only for fathers. Perceived descriptive norms explained 28.5% of the association for fathers and 37.6% for mothers; the corresponding percentages for perceived subjective norms were 9.8% and 26.6%, respectively.

**Conclusions:**

Perceived smoking norms, household smoking status, and a worksite smoking ban could be vital targets of a strategy aimed at reducing the socioeconomic disparity in parental home smoking behaviours.

## Background

Second-hand smoke (SHS) exposure is a major cause of premature death and disease in children [1]. The main source of SHS exposure for young children is parental indoor smoking [1–3]. Therefore, to protect children from SHS exposure, promoting smoke-free homes (e.g. setting voluntary rules to restrict indoor smoking at home) is the second-best option for parents who smoke, next to parental smoking cessation [4]. Younger children (e.g. aged less than 2 years) are particularly vulnerable due to their physiological features and the fact that they generally spend a lot of time with their parents [3].

The level of SHS exposure in children differs by parents’ socioeconomic status (SES) [5]. While overall prevalence of children’s SHS exposure has been significantly reduced in many developed countries [6, 7], the absence of smoke-free homes or indoor smoking behaviours among less-educated parents was 3.9 times higher than that among more-educated parents in Germany [8], 6.6 times higher in Japan [9], and 11.5 times higher in Denmark [10]. In Japan, the average prevalence of infants exposed to SHS decreased from 36.8% in 2001 to 14.4% in 2010 [9]. However, in both years, 51.5% and 28.1% of infants of less-educated parents were exposed to parental indoor smoking at home, respectively. Although evidence is scarce on the mechanisms between parental SES and indoor smoking behaviours, studies thus far have suggested that social norms of smoking play a key role in explaining SES inequality in smoking cessation [11, 12]. However, the role of these social factors in the link between parental SES and indoor smoking has not been studied.

We hypothesized that socioeconomic disparities in parental indoor smoking behaviours may arise from socioeconomic differences in individual perceptions of social norms of smoking. According to an integrated behavioural model, perceived social norms consist of descriptive and subjective norms [13]. A perceived descriptive norm is the perception of what most people do, whereas a perceived subjective norm characterizes perceived approval about performing a given behaviour by significant others, such as family and friends [13, 14]. Low-SES smokers are more likely to belong to a pro-smoking social context compared to high-SES smokers [12, 15]. If they perceive that many people around them are smoking, they may infer that smoking is common and tend to overestimate smoking prevalence (i.e. perceived descriptive norms). Subsequently, they may perceive that their significant others would approve of their smoking behaviours (i.e. perceived subjective norms) as smoking is common in their community [16]. Such a perceived acceptability of smoking predicts smoking behaviour [17], and is a vital mechanism for spreading smoking behaviour across close and distant social ties [18].

Moreover, these social norms of smoking may be affected by smoking behaviours of significant others and/or worksite smoking ban. Low-SES smokers are more likely to marry a smoker [11, 19], and mothers with a smoking partner are 7.7 times more likely to smoke indoors than mothers with a non-smoking partner [20]. Blue-collar workers are more likely to work at tobacco-friendly worksites compared with white-collar workers [21], and smoke-free workplaces reduce the prevalence of smoking among workers [22]. Therefore, having family members who smoke and/or working at tobacco-friendly worksites may be associated with indoor smoking behaviours via perceived social norms of smoking.

Emerging evidence suggest that smoke-free policies in public places and workplaces can effectively reduce tobacco use, SHS exposure, and protect child health [22–24]. Although there are legal ‘recommendations’ regarding smoke-free public places, Japan has no national legislation for comprehensive smoke-free public places. Two prefectures have an ordinance preventing SHS in public places; however, it is not comprehensively mandated in these prefectures. For workplaces, owing to the Occupational Safety and Health Act in 2014, employers are obligated to prevent SHS exposure. Although, this measure was not in effect at the time of data collection for this study, some companies had already voluntarily created smoke-free workplaces. Therefore, we used a smoke-free workplace ban as a measurement of indoor smoking policy in this study.

Consequently, we tested the mediating role of perceived social norms of smoking (perceived descriptive norms and perceived subjective norms influenced by descriptive norms) on the associations between education and indoor smoking behaviours among parents who smoke.

## Methods

This cross-sectional study was conducted using a self-administered online questionnaire through an online survey company, Macromill Inc., which is one of the largest online research panels in Japan (as of October 2014, over 2 million registered members across Japan). An online survey method is the best way to reach unique populations [25], as conventional resident registry-based random sampling has an unacceptably high cost to gather sufficient number of smoking mothers and fathers living with their young children. When compared the members of the online survey company to nationally representative populations, the distribution of main socio-demographic data (e.g. marital status, household income, and employment status) was similar [26], although the education level of participants in this study was a little higher than that of smoking parents included in the nationally representative survey [27]. Thus, the bias in this study introduced by our sampling was not expected to dramatically influence the results.

Participants were selected through convenience sampling: voluntary members of an online survey company, who met our inclusion criteria, were asked to participate. We recruited individuals who fulfilled the following inclusion criteria: (1) fathers aged 20–59 years and mothers aged 20–49 years, (2) current smokers (defined as persons who reported smoking at least 100 cigarettes in their lifetime and were currently smoking), and (3) living with one’s child who was aged 6 years or under. Considering the gender differences in the age people have children in Japan, we decided to use two different age ranges for fathers and mothers. The proportion of fathers aged 50 years or older having children in 2014 was 0.80%; however, the proportion among same-aged mothers was 0.005% [28]. We recruited fathers and mothers separately from a large sample. Although there is a possibility that some participants belong to the same couple by chance, it would not affect the results as we conducted stratified analyses by gender. We excluded individuals who were aged below 20 years (minimum legal age to smoke in Japan) since collecting information about an illegal activity was not allowed. Participants were paid through the online survey company in the form of ‘reward points’, which could be used for online shopping.

**Fig. 1:**
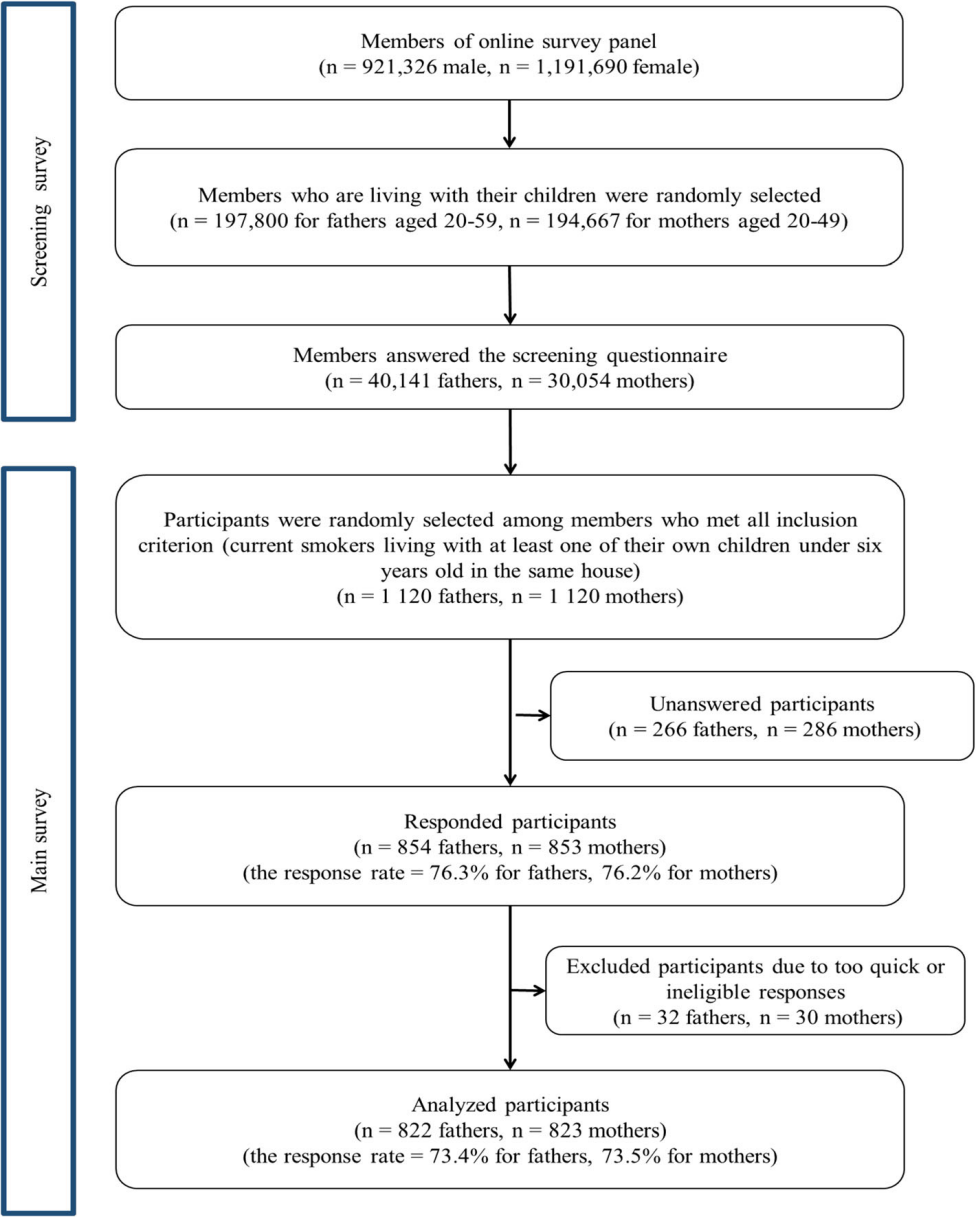
Data collection procedure

In September 2014, we collected data using a two-step process of participants screening, followed by administering the main survey (for a full description of our sampling framework, see Fig. 1). First, among the 921,326 male and 1,191,690 female panel members, 197,800 males and 194,667 females were randomly selected and received the screening survey via e-mail. Among those selected, 40,141 fathers and 30,054 mothers initially accessed the webpage and responded to the online screening questionnaire, which asked about their smoking status and the age of their children. Next, among those who met all inclusion criteria, 1120 fathers and 1120 mothers were randomly selected and invited to participate in the main survey. Then, 854 fathers and 853 mothers voluntarily accessed the webpage again and completed the online questionnaire. After excluding ineligible respondents (e.g. responded too quickly), the data from 822 fathers and 823 mothers (fathers’ response rate = 73.4%; mothers’ response rate = 73.5%) were analysed. These data cannot be made publicly available since it contains identifying information.

### Measures

#### Years of education

We calculated the years of education based on participants’ educational attainment: 9 years for middle-school graduate, 12 years for high-school graduate, 14 years for junior college or vocational college, 16 years for university graduate, and 18 years for postgraduate.

#### Indoor smoking behaviours

We assessed self-reported smoking behaviours at home with one question [29]: ‘Do you smoke cigarettes inside the house’? The response options were ‘*every day*’, ‘*more than once per week*’, ‘*more than once per month*’, ‘*several times per year*’, and ‘*never*’. We categorised smoking inside the house every day or more than once per week as indoor smoking behaviour.

#### Perceived social norms of smoking

We used six items to measure perceived descriptive norms [30], which were defined as the perception of smoking prevalence/indoor smoking prevalence of other people (including close social networks and the general public). We asked participants three items about smoking: ‘How many of your friends (or, ‘typical Japanese individuals of the same sex’, or ‘typical Japanese individuals of the same sex and from your generation’) do you think would smoke?’ We asked another three items about indoor smoking: ‘How many of your friends (or, ‘typical Japanese individual of the same sex’, or ‘typical Japanese individual of the same sex and from your generation’) do you think would smoke indoors?’ Responses were provided using a 7-point Likert scale ranging from 0% to 100% in 20-point increments (i.e. 1 = *0%*, 2 = *0–< 20%*, 3 = *20–< 40%*, 4 = *40–< 60%*, 5 = *60–< 80%*, 6 = *80–< 100%*, and 7 = *100%*). Then, we assigned the perceived descriptive norm score based on the mid-point of each prevalence category divided by 10: scores were 0 (0%), 1 (0–*<* 20%), 3 (20–*<* 40%), 5 (40–*<* 60%), 7 (60–*<* 80%), 9 (80–*<* 100%), and 10 (100%).

We used 12 items to measure perceived subjective norms [30], which were defined as the perception of whether family and friends approve of one’s own smoking behaviour. The questions were: ‘To what extent do/does your (friends/family/colleagues) approve of the following behaviours you engage in: (1) smoking, (2) smoking in your house, (3) smoking in front of others (or public places), and (4) smoking in your workplace?’ Responses were provided using a 5-point Likert scale ranging from ‘1 = *strongly disapprove*’ to ‘5 = *strongly approve*’. We asked six questions about workplace and colleagues only for parents who were employed.

For both norms, we calculated average scores for all response items; higher scores indicated higher perceptions of pro-smoking norms. The internal reliabilities (Cronbach’s alpha) were 0.87 (fathers) and 0.86 (mothers) for perceived descriptive norms, and 0.91 (fathers) and 0.93 (mothers) for perceived subjective norms.

#### Household members’ smoking status

We assessed self-reported household members’ smoking status with two questions. First, we asked about the number of cohabiting adults in the household; then, we asked if any of them smoke. We categorised the response of cohabiting with at least one household member who smoked as household members’ smoking.

#### Worksite smoking ban

We measured worksite smoking ban by asking about smoking rules in the workplace [31]. We categorised the response of worksite smoking ban with ‘complete smoking ban in the area’, ‘complete smoking ban inside the building’, ‘separation of smoking areas’, ‘no smoking ban’, and ‘no worksite’.

We also assessed participants’ age, employment status, marital status, health status in children (asthmatic or well), and age of the youngest child living at home, and adjusted for these in analysing models for both fathers and mothers.

### Statistical analyses

First, we employed bivariate regression analysis for the associations between years of education/possible mediating variables and indoor smoking behaviours using logit regression models. Then, we applied the structured equation modelling (SEM) approach to test two hypothesized models. We used Stata 13.0 (Stata Corp, College Stations, Texas) for bivariate regression analysis and M-plus 7 (Muthén & Muthén, Los Angeles, California) for all other SEM analysis. For the first model, we tested two types of social norms and household smoking status among all parents who smoked. For the second model, we added workplace smoking ban to the first model and tested it among working parents who smoked. The advantage of applying the SEM approach in this study was that it could be used to test overall models rather than individual coefficients, incorporating multiple dependents as well as mediating variables. We used the weighted least squares mean variance (WLSMV) with robust standard errors parameter estimation to estimate free parameters in the analysis, as the dependent variable (indoor smoking behaviours) was categorical [32]. Finally, we conducted mediation analysis to estimate direct and indirect (i.e. mediated) effects of education on indoor smoking behaviours using the indirect command. Regarding statistically significant indirect associations, we calculated the proportion of the indirect effect in relation to the total effect.

We tested the hypothesized model in each gender group separately. We used three model fit statistics that are commonly used in SEM: Bentler’s comparative fit index (CFI), Tucker-Lewis index (TLI), and root mean square error of approximation (RMSEA). The following model fit indices are recommended as indicating good model fit: CFI > 0.95, TLI > 0.95, and RMSEA < 0.06 [33].

The study was conducted in accordance with the World Medical Association’s Declaration of Helsinki, and this study was approved by the Research Ethics Committee of the Graduate School of Medicine at The University of Tokyo, Japan (approval number: 10603). Informed consent was obtained from all participants by clicking on an ‘I agree’ button before responding to the questionnaire.

## Results

Table 1 shows the perceived descriptive characteristics of fathers and mothers. The mean age was 38.9 years (SD = 6.8) for fathers and 31.5 years (SD = 6.0) for mothers who smoked. The mean years of education were 14.5 years (SD = 2.1) for smoking fathers and 12.8 years (SD = 2.1) for smoking mothers. Most fathers (97.9%) were employed, while over half the mothers (53.2%) were unemployed. The prevalence of indoor smoking behaviours was much higher among mothers (64.0%) than fathers (35.9%). Most fathers (78.6%) did not live with smoking household members, while over half of the mothers (65.3%) lived with at least one smoking household member.

**Table 1:** Characteristics of the study participants by gender

	Fathers who smoked (*n* = 822)		Mothers who smoked (*n* = 823)			Working fathers who smoked (*n* = 805)		Working mothers who smoked (*n* = 385)		
	Mean (n)	SD (%)	Mean (n)	SD (%)	*p*-value ^a)^	Mean (n)	SD (%)	Mean (n)	SD (%)	*p*-value ^a)^
Socio-economic status										
Years of education	14.5	2.1	12.8	2.1	< 0.001^*^	14.5	2.0	13.1	2.1	< 0.001^*^
Employment status					< 0.001^*^					
Unemployed	17	2.1	438	53.2						
Employed	805	97.9	385	46.8						
Possible mediating variables										
Perceived descriptive norms	3.8	1.8	4.1	2.1	0.005^*^	3.8	1.8	3.9	2.1	0.625*
Perceived subjective norms	3.2	0.7	3.3	0.9	0.038^*^	3.2	0.7	3.2	0.8	0.993*
Household members smoke indoors										
No or not living with an adult	646	78.6	286	34.8	< 0.001^*^	632	78.5	138	35.8	< 0.001^*^
One member smokes	122	14.8	370	45.0		119	14.8	175	45.5	
More than two members smoke	54	6.6	167	20.3		54	6.7	72	18.7	
Worksite smoking ban										
No smoking ban or no worksite						138	17.1	89	23.1	0.014^*^
Partial or complete smoking ban						667	82.9	296	76.9	
Socio-demographic status										
Age (years)	38.9	6.8	31.5	6.0	< 0.001^*^	38.9	6.8	32.2	6.1	< 0.001^*^
Age of the youngest child (years)	3.1	2.0	2.5	2.0	< 0.001^*^	3.1	2.0	2.9	1.9	0.168*
Marital status										
Unmarried	16	2.0	53	6.4	< 0.001^*^	14	1.7	40	10.4	< 0.001^*^
Married	806	98.1	770	93.6		791	98.3	345	96.6	
Smokes indoors										
No	527	64.1	296	36.0	< 0.001^*^	518	64.4	151	39.2	< 0.001^*^
Yes	295	35.9	527	64.0		287	35.7	234	60.8	

*Abbreviations*: *SD* standard deviation

*p < 0.05

^a^*p*-value is based on *t*-test for continuous variables and chi-square test for categorical variables between fathers and mothers

Binary logit regression analysis showed that, for both fathers and mothers who smoked, years of education was significantly negatively associated with indoor smoking behaviours, and the possible mediating variables were significantly positively associated with indoor smoking behaviours. For worksite smoking ban and indoor smoking behaviours, a significant negative association was found only among working fathers (Table 2).

**Table 2:** Binary logit regression estimates for indoor smoking behaviours by gender and employment status

	Fathers who smoked (*n* = 822)				Mothers who smoked (*n* = 823)				Working fathers who smoked (*n* = 805)				Working mothers who smoked (*n* = 385)			
	Smoked indoors				Smoked indoors				Smoked indoors				Smoked indoors			
	Yes	No			Yes	No			Yes	No			Yes	No		
	Mean (%)		Coef.	*p*-value	Mean (%)		Coef.	*p*-value	Mean (%)		Coef.	*p*-value	Mean (%)		Coef.	*p*-value
Socio-economic status																
Education year	14.2	14.7	−0.11	0.003^*^	12.5	13.2	−0.15	< 0.001^*^	14.2	14.7	− 0.11	0.001^*^	12.8	13.7	−0.20	< 0.001^*^
Employment status																
Unemployed	2.7	1.7	− 0.43	0.336	55.6	49.0	−0.27	0.068	N/A		N/A		N/A		N/A	
Employed	97.3	98.3			44.4	51.0										
Possible mediating variables																
Descriptive social norms	4.2	3.6	0.19	< 0.001^*^	4.4	3.5	0.21	< 0.001^*^	4.3	3.6	0.20	< 0.001^*^	4.2	3.3	0.22	< 0.001^*^
Subjective social norms	3.4	3.1	0.60	< 0.001^*^	3.4	3.0	0.58	< 0.001^*^	3.4	3.1	0.64	< 0.001^*^	3.3	3.0	0.45	0.001^*^
Household members smoke indoors																
No or not living with an adult	69.5	83.7	0.49	< 0.001^*^	32.1	39.5	0.21	0.035^*^	69.3	83.6	0.49	< 0.001^*^	31.6	42.4	0.35	0.020^*^
One member smokes	22.0	10.8			46.3	42.6			22.0	10.8			47.0	43.1		
More than two members smoke	8.5	5.5			21.6	17.9			8.7	5.6			21.4	14.6		
Worksite smoking bans																
No smoking bans or no worksite	N/A		N/A		N/A		N/A		21.3	14.9	−0.44	0.022^*^	24.8	20.5	−0.24	0.334
Partial or complete smoking bans									78.8	85.1			75.2	79.5		
Socio-demographic status																
Age (years)	39.6	38.5	0.02	0.032^*^	31.2	32.0	−0.02	0.063	39.6	38.5	0.02	0.039^*^	31.5	33.2	−0.05	0.008^*^
Age of the youngest child (years)	3.5	2.8	0.18	< 0.001^*^	2.5	2.4	0.01	0.761	3.6	2.8	0.18	< 0.001^*^	2.8	3.1	−0.09	0.103^*^
Marital status																
Unmarried	3.4	1.1	−1.11	0.033^*^	7.2	5.1	−0.38	0.232^*^	3.1	1.0	−1.20	0.033^*^	12.4	7.3	−0.59	0.113^*^
Married	96.6	98.9			92.8	94.9			96.9	99.0			87.6	92.7		

*Abbreviations*: *N/A* not applicable

^*^p < 0.05

**Fig. 2:**
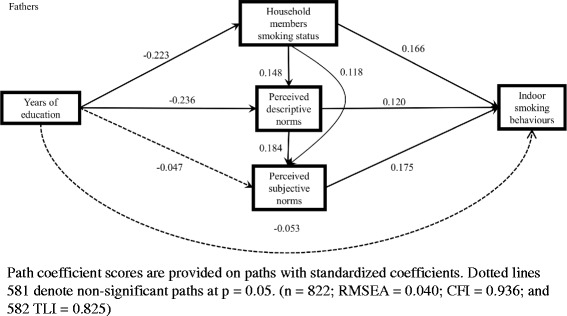
Model of the pathways between education and indoor smoking behaviours among fathers who smoked

**Fig. 3:**
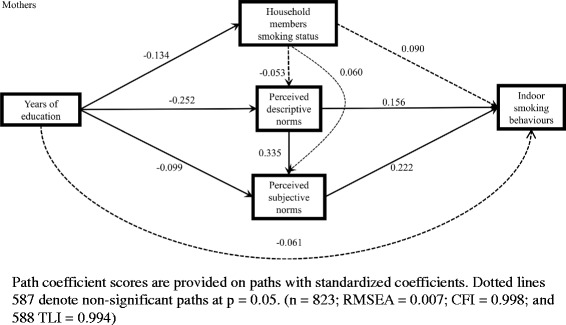
Model of the pathways between education and indoor smoking behaviours among mothers who smoked

Figures 2 and 3 show the standardized coefficients in the model among fathers and mothers. For fathers who smoked, years of education was negatively associated with perceived descriptive norms of smoking, whereas descriptive norms and following subjective norms were positively associated with indoor smoking behaviours. Household smoking was positively associated with each social norm of smoking, and mediated the association between education and indoor smoking behaviours. The indirect effect of education on indoor smoking via either perceived descriptive or subjective norms of smoking was significant (coef. = − 0.046; proportion of the total effect = 32.0%) (Table 3).

**Table 3:** Standardized estimates of total, total indirect, specific indirect, and direct effects

Among fathers who smoke	Estimate	*P*-value	The proportion to the total effect
Total	−0.144^*^	0.001	
Direct	−0.053	0.228	
Total indirect	−0.091^*^	< 0.001	63.2%
Specific indirect			
education→household members smoking→indoor smoking	−0.037^*^	0.013	25.7%
education→descriptive norms→indoor smoking	−0.028^*^	0.011	19.4%
education→subjective norms→indoor smoking	−0.008	0.208	
education→household members smoking→descriptive norms→indoor smoking	−0.004^*^	0.046	2.8%
education→household members smoking→subjective norms→indoor smoking	−0.005^*^	0.047	3.5%
education→descriptive norms→subjective norms→indoor smoking	−0.008^*^	0.003	5.6%
education→household members smoking→descriptive norms→subjective norms→indoor smoking	−0.001^*^	0.028	0.7%
Among mothers who smoke	Estimate	*P*-value	The proportion to the total effect
Total	−0.154^*^	0.001	
Direct	−0.061	0.175	
Total indirect	−0.092^*^	< 0.001	59.7%
Specific indirect			
education→household members smoking→indoor smoking	−0.012	0.097	
education→descriptive norms→indoor smoking	−0.039^*^	0.002	25.3%
education→subjective norms→indoor smoking	−0.022^*^	0.013	14.3%
education→household members smoking→descriptive norms→indoor smoking	0.001	0.213	
education→household members smoking→subjective norms→indoor smoking	−0.002	0.132	
education→descriptive norms→subjective norms→indoor smoking	−0.019^*^	< 0.001	12.3%
education→household members smoking→descriptive norms→subjective norms→indoor smoking	0.001	0.19	
Among working fathers who smoke	Estimate	*P*-value	The proportion to the total effect
Total	−0.153^*^	< 0.001	
Direct	−0.040	0.429	
Total indirect	−0.112^*^	0.001	73.2%
Specific indirect			
education→household members smoking→indoor smoking	−0.035^*^	0.017	22.9%
education→descriptive norms→indoor smoking	−0.022^*^	0.023	14.4%
education→subjective norms→indoor smoking	−0.010	0.215	
education→workplace smoking policies→indoor smoking	−0.020	0.491	
education→household members smoking→descriptive norms→indoor smoking	−0.004^*^	0.045	2.6%
education→workplace smoking policies→descriptive norms→indoor smoking	−0.007^*^	0.046	4.6%
education→household members smoking→subjective norms→indoor smoking	−0.005^*^	0.043	3.3%
education→desriptive norms→subjective norms→indoor smoking	−0.006^*^	0.008	3.9%
education→workplace smoking policies→subjective norms→indoor smoking	< 0.001	0.948	
education→household members smoking→desriptive norms→subjective norms→indoor smoking	−0.001^*^	0.028	0.7%
education→workplace smoking policies→descriptive norms→subjective norms→indoor smoking	−0.002^*^	0.041	1.3%
Among working mothers who smoke	Estimate	*P*-value	The proportion to the total effect
Total	−0.234^*^	< 0.001	
Direct	−0.140	0.055	
Total indirect	−0.094^*^	< 0.001	40.2%
Specific indirect			
education→household members smoking→indoor smoking	−0.028	0.063	
education→descriptive norms→indoor smoking	−0.035^*^	0.026	15.0%
education→subjective norms→indoor smoking	−0.024	0.104	
education→workplace smoking policies→indoor smoking	< 0.001	0.97	
education→household members smoking→descriptive norms→indoor smoking	0.002	0.261	
education→workplace smoking policies→descriptive norms→indoor smoking	−0.003	0.199	
education→household members smoking→subjective norms→indoor smoking	< 0.001	0.866	
education→desriptive norms→subjective norms→indoor smoking	−0.006	0.109	
education→workplace smoking policies→subjective norms→indoor smoking	−0.002	0.287	
education→household members smoking→desriptive norms→subjective norms→indoor smoking	< 0.001	0.303	
education→workplace smoking policies→descriptive norms→subjective norms→indoor smoking	< 0.001	0.252	

^*^p < 0.05

For mothers who smoked, both perceived descriptive and subjective norms of smoking mediated the association between education and indoor smoking behaviours. However, household smoking was neither significantly associated with social norms of smoking nor indoor smoking behaviours. The indirect effect of education on indoor smoking behaviours via either perceived descriptive or subjective norms of smoking was significant (coef. = − 0.080; proportion of the total effect = 51.9%) (Table 3).

**Fig. 4:**
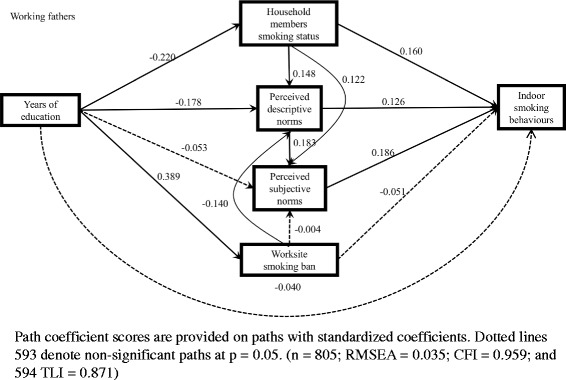
Model of the pathways between education and indoor smoking behaviours among working fathers who smoked

**Fig. 5:**
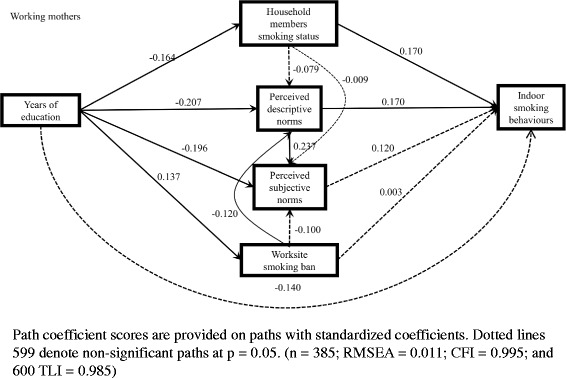
Model of the pathways between education and indoor smoking behaviours among working mothers who smoked

Figures 4 and 5 show the standardized coefficients in the model among working fathers and mothers who smoked. For fathers, worksite smoking ban was positively associated with perceived descriptive norms of smoking, and the indirect effect of education on indoor smoking via worksite smoking ban and perceived descriptive norms of smoking was significant (coef. = − 0.009, 5.9%) (Table 3). For mothers, worksite smoking ban was positively associated with perceived subjective norms of smoking; however, the indirect effect via worksite smoking ban was not significant (Table 3).

For the above four models, WLSMV estimation showed acceptable model fit values, and the direct association between education and indoor smoking was not statistically significant.

## Discussion

Perceived social norms of smoking mediated the association between education and indoor smoking behaviours among parents who smoke. Further, only for fathers, household smoking status and worksite smoking ban mediated the association between education and indoor smoking via perceived norms of smoking. Social norms of smoking is suggested to be a mechanism between SES and smoking behaviours [34], and this study extended evidence that social norms explain the associations between education and indoor smoking behaviours among parents who smoke.

As hypothesized, perceived descriptive and subjective norms of smoking mediated the pathways between education and indoor smoking behaviours. According to a study that examined the person-to-person spread of smoking behaviours [18], smokers in interconnected groups (such as partners, siblings, and friends) shared social norms of smoking and changed their smoking behaviours (such as quitting smoking) together. As the prevalence of smoking is concentrated in lower-SES groups in Japan, smoking parents with less formal education may share an overestimation of smoking prevalence (perceived descriptive norm) and greater acceptability of smoking (perceived subjective norm) in their social networks. Therefore, they may tend to continue indoor smoking behaviours, while parents with more formal education are more likely to stop these behaviours due to social pressure [35]. This result suggests that overestimation and greater acceptability of smoking would be an acceptable explanation of such disparity in Japan.

The social norms of smoking were associated with environmental variables (worksite smoking and household smoking bans) among fathers who smoked, but not among mothers who smoked. As less-educated fathers are more likely to work in places without worksite smoking ban, they tend to have a greater acceptance of smoking (perceived subjective norms) through overestimating smoking prevalence (perceived descriptive norms), and leading to higher indoor smoking. Previous studies suggest that smoke-free policies lower the perceived acceptability of tobacco exposure, even among smokers [36], with decreased acceptability leading to a reduction in tobacco use [37]. In addition, the acceptability of smoking in a work unit is considered to impact worksite smoking policies on smoking cessation [38]. The present study suggested that smoking fathers may also increase the awareness of the need to prevent SHS exposure at home when they feel that smoking is not accepted in their workplaces; therefore, they are more likely to stop smoking at home even if they do not quit smoking. Similarly, for fathers who smoke, cohabiting with family members who smoke results in a consensus about what is acceptable, and may motivate them to engage in the same behaviours. Alternatively, however, as this was a cross-sectional study, an effect in the opposite direction may also be possible (e.g. individuals with similar smoking norms may tend to marry each other or work in similar places). Future studies should collect longitudinal data to identify more accurate causal pathways.

A unique finding of this study was the gender difference in the associations between social norms of smoking and environmental variables. A study in the United States found that women were twice as likely to report social pressure to quit smoking compared to men [35]. However, in this study, household smoking was not associated with perceived norms of smoking for mothers. One possible explanation is that mothers’ smoking norms may be influenced by friends’ behaviours, rather than families or colleagues. Alternatively, mothers’ smoking norms may be under more pressure from a much wider social context than those of fathers [39]. Traditional norms about female gender roles may cause pressure of not to smoke for women [40]. Simultaneously, because of tobacco marketing women have been exposed to an institutional gender norm that those who smoke are feminine, free, and stylish [41].

These findings provide new insights for effective interventions to narrow the educational disparities in parental indoor smoking behaviours. This study suggests that less- and more-educated smokers perceive social norms of smoking differently in their distinct social networks. This may be one of the reasons why less-educated smokers are less responsive to smoke-free legislation than more-educated smokers [42]. Therefore, it may be effective to modify perceived pro-smoking norms in less-educated fathers by encouraging smoking cessation for household members including grandparents and others who smoke, and by promoting workplace smoking ban especially for blue-collar workplaces or small companies where less-educated fathers tend to work [43].

This study has several limitations. First, since we employed a cross-sectional design, we cannot infer causality. Although educational attainment would not have changed over the time of interest, indoor smoking behaviour could influence household members’ smoking status. Second, selection bias might have existed due to the self-selection of participants in the online survey. As the participants had higher levels of education than nationally representative random samples, the association between education level and mediating variables might have been underestimated. However, as the distribution of main socio-demographic data among online survey panel members are similar to those found in nationally representative random samples, we believe that our data are generalizable to smoking parents with young children in Japan. Third, other critical and omitted variables might exist that may affect the mediating role of social norms of smoking such as peers’ smoking behaviours, and social norms of smoking at the community level (not perceived norms at the individual level) [44].

Despite these limitations, this study is the first to examine the multiple pathways between education and indoor smoking behaviour, using an SEM approach, among parents who smoke with young children. Our results aid in the understanding of the mechanisms in this relationship to a greater degree than would have been achieved by a general multiple regression analysis. Further, stratified analysis of fathers and mothers allowed for more accurate inferences about the gender specificity of the pathways.

## Conclusions

In conclusion, this study provided evidence that parents who smoked with less formal education were more likely to perceive pro-smoking descriptive and subjective norms and these norms were significantly associated with their indoor smoking behaviours. In addition, for fathers who smoked, smoking among colleagues and household members had an indirect effect on indoor smoking behaviours via perceived norms of smoking. Discouraging pro-smoking norms in home and work social networks could help to reduce indoor smoking behaviours and narrow socioeconomic disparities in children’s SHS exposure.

## Abbreviations

CFI: Comparative fit index
RMSEA: Root mean square error of approximation
SEM: Structured equation modelling
SES: Socioeconomic status
SHS: Second-hand smoke
TLI: Tucker-Lewis index
WLSMV: Weighted least squares mean variance
